# Parenting stress, dyadic coping and endocrine markers of stress and resilience in foster and biological mothers

**DOI:** 10.1371/journal.pone.0310316

**Published:** 2024-09-10

**Authors:** Vanessa Reindl, Arnold Lohaus, Nina Heinrichs, Kerstin Konrad

**Affiliations:** 1 Medical Faculty, Department of Child and Adolescent Psychiatry, Child Neuropsychology Section, Psychosomatics and Psychotherapy, RWTH Aachen University, Aachen, Germany; 2 JARA-Brain Institute II, Molecular Neuroscience and Neuroimaging, RWTH Aachen & Research Centre Juelich, Aachen, Germany; 3 Psychology, School of Social Sciences, Nanyang Technological University, Singapore, Republic of Singapore; 4 Department of Psychology, Bielefeld University, Bielefeld, Germany; 5 Department of Psychology, Institute of Clinical Psychology, Psychotherapy and Assessment, University of Braunschweig, Braunschweig, Germany; 6 Department of Clinical Child and Adolescent Psychology and Psychotherapy, Bielefeld University, Bielefeld, Germany; Universitat der Bundeswehr München: Universitat der Bundeswehr Munchen, GERMANY

## Abstract

Foster parents have been shown to report higher levels of parenting stress but also more dyadic coping (DC) behaviors in their partnership than biological parents, which might be an important protective factor that helps them cope with daily stressors. Here, we examined how parenting stress and DC are related in foster and biological parents and whether these are reflected in long-term alterations of hypothalamic-pituitary-adrenocortical (HPA) axis activity. A total of 79 foster mothers and 131 biological mothers participated in a longitudinal study. At the initial assessment, children were aged 2–7 years and lived for an average of 18 months in their current foster family. Mothers’ cortisol and dehydroepiandrosterone (DHEA) concentrations and their cortisol/DHEA ratios were assessed in scalp hair twice with approximately 11 months in between, while their perceived parenting stress and DC were measured by self-report questionnaires. Results showed no significant differences between foster mothers and biological mothers in cortisol, DHEA and cortisol/DHEA concentrations. While more DC was longitudinally related to lower levels of parenting stress across both study groups, no significant associations were found to endocrine markers. Thus, these findings indicate that increased parenting stress levels were not, or not strongly, reflected in HPA axis alterations as assessed in hair. Our findings thus add evidence for non-significant associations between self-reported perceived stress and chronic HPA axis markers. Future studies may explore whether early interventions, including those aimed at promoting and maintaining positive DC, are beneficial in preventing the development of stress-related illnesses in foster parents.

## 1. Introduction

Many children in foster care have experienced persistent maltreatment, such as abuse and neglect, which is the most common reason for out-of-home placement and is associated with an increased risk of developing mental health problems [1]. Behavioral problems require additional support and supervision from foster parents, in addition to the challenges of developing new emotional attachments and family routines after the child has entered the foster family. As a result, foster parents face increasing parenting demands that can cause parenting stress. Consistent with this, we found increased internalising (symptoms directed inwards, e.g., sadness) and externalising (symptoms directed outwards, e.g., aggressive behavior) problems in 2–7 year old children in foster care which were accompanied by increased parenting stress in foster compared to biological parents and remained relatively stable over a period of 12 months (using the same sample as in the current study [2,3]). Parenting stress, as a specific form of stress associated with raising children, has been shown to be positively related to overall stress perception, and negatively to psychological well-being (e.g., anxiety, guilt) and the quality of other family relationships (e.g., lower marital satisfaction [4]).

Both parents’ stress levels and their coping behaviors may be strongly intertwined and can directly or indirectly affect each other in a mutual way (see ‘systemic transactional model of stress’ [5]). In this context, dyadic coping (DC) describes the way parents jointly cope with daily hassles [5]. It can manifest both in positive ways, e.g., showing empathy, taking over tasks and duties of the partner or searching for solutions together, and in negative ways, e.g., blaming and criticizing the partner or offering ambivalent support. Thus, positive DC may allow foster parents to more effectively deal with challenging child conditions, thereby reducing overall stress levels and parenting stress in particular. A recent study reported high levels of positive DC and low levels of negative DC in Italian prospective adoptive parents, indicating that adoptive couples were particularly well-equipped with respect to relationship resources and couple relationship functioning, however a direct comparison group was missing [6]. In line with this, we found better overall DC (aggregated score after reversing negative items) in foster parents compared to biological parents approximately 18 months after foster care placement (using the same sample as in the current study [7]). While the relationship between DC and parenting stress has not been examined in foster parents so far, a negative association has been found for parents of children with autism spectrum disorder (ASD) at a single point in time. However, the directionality of effects is unclear [8].

By increasing or decreasing overall stress levels, respectively, parenting stress and DC may affect the parent’s biological functioning. The hormones cortisol and dehydroepiandrosterone (DHEA) are both released by the hypothalamic-pituitary-adrenocortical (HPA) axis as outputs of a coordinated hormonal cascade to stress (specifically, 100% of the body’s cortisol and ~ 80% of DHEA are produced by the adrenals [9]). Given that DHEA and its sulphated metabolite DHEA-S may antagonize some of the effects of cortisol, the unopposed effects of cortisol, reflected in higher cortisol/DHEA(-S) ratios, is traditionally assumed to be associated with greater stress-related mental or physical health risks [9]. While these hormones are most commonly sampled in saliva, blood or urine, reflecting the hormone concentrations at the time of collection, more recent methods allow the quantification of hormone concentrations in human hair as a strategy for assessing cumulative hormone concentrations over extended periods of time (e.g., three months). However, no study that we know of has examined cortisol and DHEA concentrations in foster parents (irrespective of measurement modality) and their relation to perceived parenting stress and DC.

Although cortisol and DHEA have been proposed as biomarkers of stress and resilience, i.e., the ability to positively adjust to adverse experiences, past studies have produced controversial results [10]. Findings suggest that hair cortisol levels may be elevated in subjects with ongoing exposure to chronic stress, e.g., in dementia caregivers [11], long-term unemployed individuals [12] or parents with parental burnout [13]. However, when it comes to self-reported stress levels and social support, a meta-analysis reported no overall effect [14]. Few studies have investigated (hair) cortisol concentrations in parents who care for a child with a neurodevelopmental disorder but also with contradictory results [15–17].

For DC there is some evidence that it reduces stress as marked by decreasing salivary cortisol levels in community samples. For instance, it has been shown that partners’ positive DC behaviors accelerated the rate at which stressed individuals’ salivary cortisol levels recovered following psychosocial stress elicited by the Trier Social Stress Test (TSST; [18]). Further, partner responsiveness predicted diurnal cortisol profiles, i.e., steeper cortisol slopes, 10 years later [19]. Yet, other studies found no relationship between DC and salivary cortisol levels [20].

Much less research has been conducted on DHEA(-S) and cortisol/DHEA(-S) ratios. On the one hand there is evidence that chronic or prolonged stress is associated with attenuated DHEA-S levels (in subjects who reported stress at work, measured in serum [21]) and lower DHEA to cortisol ratios (in high-stress compared to low-stress subjects living with HIV, measured in hair [22]). On the other hand, a rise in DHEA levels during stress may be related to resilience. For instance, a significant rise in salivary DHEA, with large inter-individual variation, was found in healthy subjects in response to the TSST and was positively correlated to the subjects’ cortisol responses [23,24]. Higher DHEA levels and lower cortisol/DHEA ratios during TSST were associated with less negative mood during and after the TSST [23]. Consistently, measures of resilience (sense of coherence and self-care) have been associated with higher DHEA levels and lower cortisol/DHEA ratios, measured in hair, in professional caregivers in youth residential care institutions [25]. While this group of subjects may also experience increased levels of caregiving stress, no study that we know of has examined DHEA in foster parents and in relation to parenting stress or DC.

Taken together, it is currently unclear how perceived parenting stress and DC in the partnership influence each other over time in foster and biological parents and how these are related to endocrine stress markers. In the current study, we therefore asked: 1) How are perceived parenting stress and DC in the partnership related in foster and biological mothers? 2) Do foster mothers show differences in hair cortisol and DHEA concentrations and cortisol/DHEA ratios compared to biological mothers?, and 3) How are perceived parenting stress and DC related to endocrine markers? If indeed perceived parenting stress is reflected by long-term changes in the HPA axis, foster mothers are expected to have higher cortisol levels, lower DHEA levels as well as higher cortisol/DHEA ratios. Yet, such potential changes may be counteracted by more positive DC behavior in foster mothers. Given findings that hair DHEA is positively correlated to resilience in youth residential caregivers [25], higher DC in foster mothers may be related to higher DHEA levels and lower cortisol/DHEA ratios. Thus, in the current study, both the individual and interactive contributions of parenting stress and DC to hormone concentrations were examined.

## 2. Methods

### 2.1 Sample

Data were collected as part of a larger project between April 2014 and March 2017 investigating the development of children in foster care and the effects of an intervention (for more information see https://www.uni-bielefeld.de/fakultaeten/psychologie/abteilung/arbeitseinheiten/20/forschung/abgeschlossene-forschungs/grow-treat/index.xml). Foster families were mainly recruited via youth welfare offices in three German regions. Youth welfare offices were asked to contact long-term, non-kinship care foster families with children aged two to seven years who had been in their current foster family for ≤ 24 months and had a history of abuse and/or neglect (for an analysis of the types of maltreatment experienced see [26]). Biological families were recruited in the same age range mostly via postings or parents’ evenings in nursery and elementary schools. Informed consent was provided by the foster parents and the person(s) holding child custody for the participation of the foster families. In case of biological families, informed consent was provided by the biological parents. Families received a monetary award for their participation. The study was approved by the ethics committee of the German Society for Psychology and additionally by the Medical Faculty of the RWTH Aachen (EK 096/14) for the hair steroid hormone measures.

The longitudinal part of the project consisted of three measurement time points (T1, T2 and T3) in six-month intervals (T1 to T2: *M* = 6.38 months, *SD* = 1.54 months; T2 to T3: *M* = 5.98 months, *SD* = 1.45 months). After T1, approximately half of the foster parents were offered to take part in a parent group training [27]. The training had no effects on any of its primary or secondary outcome measures (which did not include any of the presently targeted study variables [27]). Since we did also not find any effects on hair steroid hormones, DC and parenting stress (see S1 Table), the intervention is not further considered in the analyses.

A total of 94 children in foster care (foster care group, FC) and 157 biological children (biological control group, BC) participated in the study at T1. From T1 to T3, 10 children in foster care and 11 biological children dropped out of the study. In few cases, one (foster) parent participated with more than one (foster) child, in this case, one child was randomly selected. Hair steroid hormone data was collected of the primary caregiver which was either the (foster) mother or (foster) father. However, since for the great majority of children, the (foster) mother was the primary caregiver and to reduce gender effects on hormone analysis, only data of the (foster) mothers were reported in the manuscript, which led to the exclusion of ten subjects. Further, for some mothers no hair steroid hormone data was available at any of the assessments (e.g., due to insufficient amounts, outliers) and two mothers were excluded due to endocrine disorders. Thus, the final sample for the current analyses consisted of 79 foster mothers and 131 biological mothers, who had valid hair steroid hormone data for at least one assessment. All missing values were imputed (see 2.2.1 for more information on missing values and 2.3 for analysis). At T1, children in foster care lived on average 17.62 months (*SD* = 8.94 months) in their foster families. The two groups did not differ with respect to child gender, family socioeconomic status (SES) and relationship status of the parents (see Table 1 for a sample description). However, foster mothers were significantly older than biological mothers and children in foster care were significantly younger than biological children. As a consequence, all statistical analyses included mother’s age and child’s age as control variables. Further, foster parents reported significantly more emotional and behavioral problems of their children compared to biological parents, whereby *n* = 22 (29.3%) of the children in foster care and *n* = 16 (12.7%) of the biological children had a Child Behavior Checklist (CBCL) total score *T* value ≥ 60 at T1, indicating scores in the borderline clinical (*T* values: 60–63) or clinical range (*T* values > 63).

**Table 1:** Sample description and comparison between the foster care group and biological control group.

	BC	FC	Test Statistic
Child’s age in years, T1 (*M*, *SD*)	4.51 (1.44)	3.75 (1.56)	*t* (208) = 3.586, *p* < .01, *d* = -.51
Child’s gender (female: *n*, %)	69 (52.7%)	40 (50.6%)	*χ*^*2*^ (1, 210) = .082, *p* = .78
Mother’s age in years, T1 (*M*, *SD*)	35.82 (5.33)	41.05 (6.71)	*t* (136.78) = - 5.904, *p* < .01, *d* = .89
Mothers married / living in a stable relationship (n, %)	120 (93.0%)	74 (93.6%)	*χ*^*2*^ (2, 208) = 1.312, *p* = .52
SES, T1 (*M*, *SD*)	13.59 (4.01)	13.94 (3.69)	*t* (204) = -.617, *p* = .27
Mother’s BMI (*M*, *SD*)			*F* (1, 130) = 4.775, *p* = .03, *η*^*2*^ = .035
T1	24.86 (5.04)	27.19 (6.92)	
T3	24.64 (5.03)	27.23 (6.71)	
CBCL (*M*, *SD*)			*F* (1, 183) = 9.253, *p* < .01, *η*^*2*^ = .048
T1	49.98 (9.84)	53.25 (10.46)	
T3	46.54 (9.13)	51.81 (11.10)	

*Note*. BC: Biological control group. FC: Foster care group. Relationship status of mothers was coded as 1 = married, 2 = living in a stable relationship and 3 = single. SES: Socioeconomic status. BMI: Body Mass Index. CBCL: Total score of the Child Behavior Checklist (*T*-value). Sample description is based on data prior to multiple imputation.

Hair steroid hormone data of the mother was collected at the first and the third assessment. In a few cases, data was not available at T1. In these cases (*n* = 25), data were collected at T2 instead and used to replace the T1 data for the current analyses (collectively referred to as T1 in the analyses and results). T1 and T2 data did not significantly differ with respect to hormone concentrations, parenting stress or DC. Further, all findings of the manuscript were replicated without replacing missing T1 measurements by T2. For all analyses, questionnaire data of the same time point (T1 or T2) as the hormone assessments were considered. The time between T1 and T3 was *M* = 11.37 months (*SD* = 2.53 months).

### 2.2. Variables

#### 2.2.1 Maternal hair steroid hormones

Two to three hair strands were collected from the posterior vertex region of the head and stored in aluminum foil until further analysis (as described in [28]). Cortisol and DHEA concentrations were determined in the first scalp-near 3 cm hair segment, reflecting the cumulative hormone secretion of the past three months, and analyzed using liquid chromatography-tandem mass spectrometry (LC-MS/MS; [29]) at TU Dresden at the end of the study. In most cases, 7.5 mg of whole, non-pulverized hair was used for LC-MS/MS analyses, samples with less than 4 mg are of insufficient amount for hair steroid hormone analysis and were thus discarded (T1: *n* = 19, T2: *n* = 2, T3: *n* = 7).

Cortisol/DHEA values were derived by dividing cortisol values by DHEA values. Since values showed positively skewed distributions, cortisol, DHEA, cortisol/DHEA values were log-transformed and afterwards outlying values (± 3 SDs from the mean) were excluded (cortisol: *n* = 3, DHEA: *n* = 4, cortisol/DHEA: *n* = 2). Raw values are depicted in S2 Table.

Detailed information on potential confounding variables for maternal hair steroid hormones, including hair treatments, hair washing frequency, hair color, chronic diseases, cortisone medication intake, oral contraceptive intake, smoking and Body Mass Index (BMI), was available in a subsample of *n* = 142 participants. Foster care and comparison group differed only with respect to BMI (higher mean BMI in foster mothers; Table 1) and intake of oral contraceptive (more commonly used in biological mothers, possibly related to younger mean age; at T3: *χ²* (1, *N* = 134) = 7.177, *p* = .008). Intake of oral contraceptive was not related to cortisol or DHEA. However, DHEA was significantly associated with BMI, so BMI was controlled for in all subsequent analyses including DHEA. Of note is that while additionally progesterone concentrations were acquired, these were not analysed since progesterone was strongly related to intake of oral contraceptives.

#### 2.2.2 Questionnaires

**Parenting stress** of (foster) mothers was assessed by the Parental Stress Questionnaire [30] at T1, T2, and T3 on a 17-item parental distress subscale. Example items are “I struggle a lot with my child” and “Sometimes I feel insecure in raising my child”. All items were answered on a 4-point rating scale (0 = “strongly disagree”, 1 = “disagree”, 2 = “agree”, and 3 = “strongly agree”) with respect to how they are “typically” perceived, and sum scores were calculated for the subscale. We administered either a preschool or a school version of the questionnaire, depending on whether the child already attended school or not. The subscale values of the two versions were z-standardized (across the two groups, but separately for the preschool and school versions) and all analyses were based on z-values. The internal consistencies of the subscale in our sample ranged between *α* = .88 (T1, school) and *α* = .94 (T2, school).

**Dyadic coping** of the (foster) mothers was assessed at T1, T2 and T3 by the total score of the Dyadic Coping Inventory (DCI; [31]), which is designed to measure how couples communicate stress and cope with common everyday stressors. The DCI measures DC as a multidimensional construct including supportive, delegated and negative DC by self and partner as well as joint (common) DC. Example items are “I ask my partner to do things for me when I have too much to do” and “We try to cope with the problem together and search for ascertained solutions”. Here, an overall DC score was calculated based on 35 items, assessed on 5-point rating scales (1 = “very rarely” to 5 = “very often”). For the current analyses, we used the mean rating across all 35 items after reversing the negative DC items, allowing for 20% missing values (see [7]). Internal consistencies ranged between *α* = .92 (T3) and *α* = .93 (T1).

For sample characterization, child’s **emotional and behavioral problems** were assessed (at T1, T2, T3) by parent-reports using *T* scores for the total score of the German versions of the CBCL (CBCL 1½–5 for children aged 2–4 years [32]; CBCL 4–18 for children aged 5–9 years [33]). Further, **family SES** was assessed at T1 based on the social class index by [34] and potential confounding variables for maternal hair steroid hormones (see 2.2.1) were measured in a self-developed interview.

### 2.3 Statistical analyses

Data analyses were conducted in IBM SPSS Statistics 27 and R version 3.6.2 [35]. For the R packages used and their implementation details, see S1 Text. Values of *p* < 0.05 (two-tailed) were considered statistically significant. False discovery rate (FDR) correction of *p*-values was performed for each research question and analysis type (*p*_*adj =*_ FDR adjusted *p*-values; *p* = unadjusted *p*-values; see S1 Text).

A non-significant Little’s MCAR test indicated that hair steroid, parenting stress and DC data were missing completely at random (*p* = .88). Prior to statistical analyses, missing values were imputed using multiple imputation (*N* = 20) and imputed data sets were merged into a single data set in which each missing value was replaced by the mean of all imputed values (S1 Text).

First, to explore the relationships between parenting stress and DC (research question 1), we calculated a total of four partial Pearson correlations for the two measurement time points and two groups separately controlling for mother’s age (T1) and child’s age (T1 or T3, respectively). To further test the longitudinal relationships, we used hierarchical regression analyses to examine whether DC at T1 significantly predicted parenting stress at T3 or vice versa. To this end, mother’s age, child’s age, group and parenting stress at T1 were included as predictors in step 1, DC at T1 in step 2 and the interactive term of DC and group in step 3. Parenting stress at T3 was the dependent variable. Regressions predicting DC at T3 were conducted analogously with DC at T1 as a first step and parenting stress at T1 as a second step predictor. Continuous variables were mean-centered. The T1 value of the dependent variable was included as a first step predictor to capture the effect of DC on parenting stress (and vice versa), taking the respective baseline level into account. In addition, group differences in parenting stress and DC were calculated via linear mixed models (LMMs). These have been reported previously [2,3,7], but were re-analyzed in the current manuscript for the sake of completeness with a reduced sample size due to missing hair steroid hormone data.

Second, to examine differences in hair steroid hormones (research question 2), we calculated three separate LMMs with cortisol, DHEA and cortisol/DHEA as dependent variables, respectively (see S1 Text). The full model included a random intercept for subjects and the fixed effects of measurement time and group (0 = biological mothers, 1 = foster mothers) as well as child’s age (at T1, in years) and mother’s age (at T1, in years) as control variables. Further, for the model examining differences in DHEA, mother’s BMI was included as an additional control variable. Time was coded as a continuous variable. For T1, this value was always 0 and for T3 it corresponded to the time between the T1 and T3 measurement time points (see also [28]). We defined time as a continuous instead of categorical variable since the time between T1 and T3 varied. Again, continuous variables were mean-centered. Second, to test for any differential change over time between the two groups, the time x group interaction was added as a predictor to the LMMs described above. For nonsignificant group differences, we calculated the Bayes factor (B) to test the relative strength of evidence for the null hypothesis, i.e., there are no differences between foster and biological mothers in hair steroid hormone concentrations, and for the alternative hypothesis, i.e., foster and biological mothers differ in hormone concentrations (see S1 Text). B can vary between 0 and infinity, whereby 1 indicates that the data does not favor one theory more than the other and values close to 0 speak in favor of the null hypothesis [36]. To interpret the findings, we used the cut-offs suggested by [37]: B < 1/3 and B > 3 represent substantial evidence for the null and alternative hypothesis, respectively, while B values between 1/3 and 3 represent only weak or “anecdotal” evidence.

Third, to examine the relationships between hair steroid hormones, parenting stress and DC (research question 3), we calculated a series of partial Pearson correlations for the two measurement time points and two groups separately controlling for mother’s age (T1), child’s age (T1 or T3, respectively) and, in analyses with DHEA, mother’s BMI (24 correlations in total). The effects were then tested by adding i) the main effects of parenting stress and DC and ii) the main and interactive effects of parenting stress and DC in separate models to the LMMs predicting cortisol, DHEA and cortisol/DHEA.

## 3. Results

### 3.1 Relationship between parenting stress and DC

**Fig 1:**
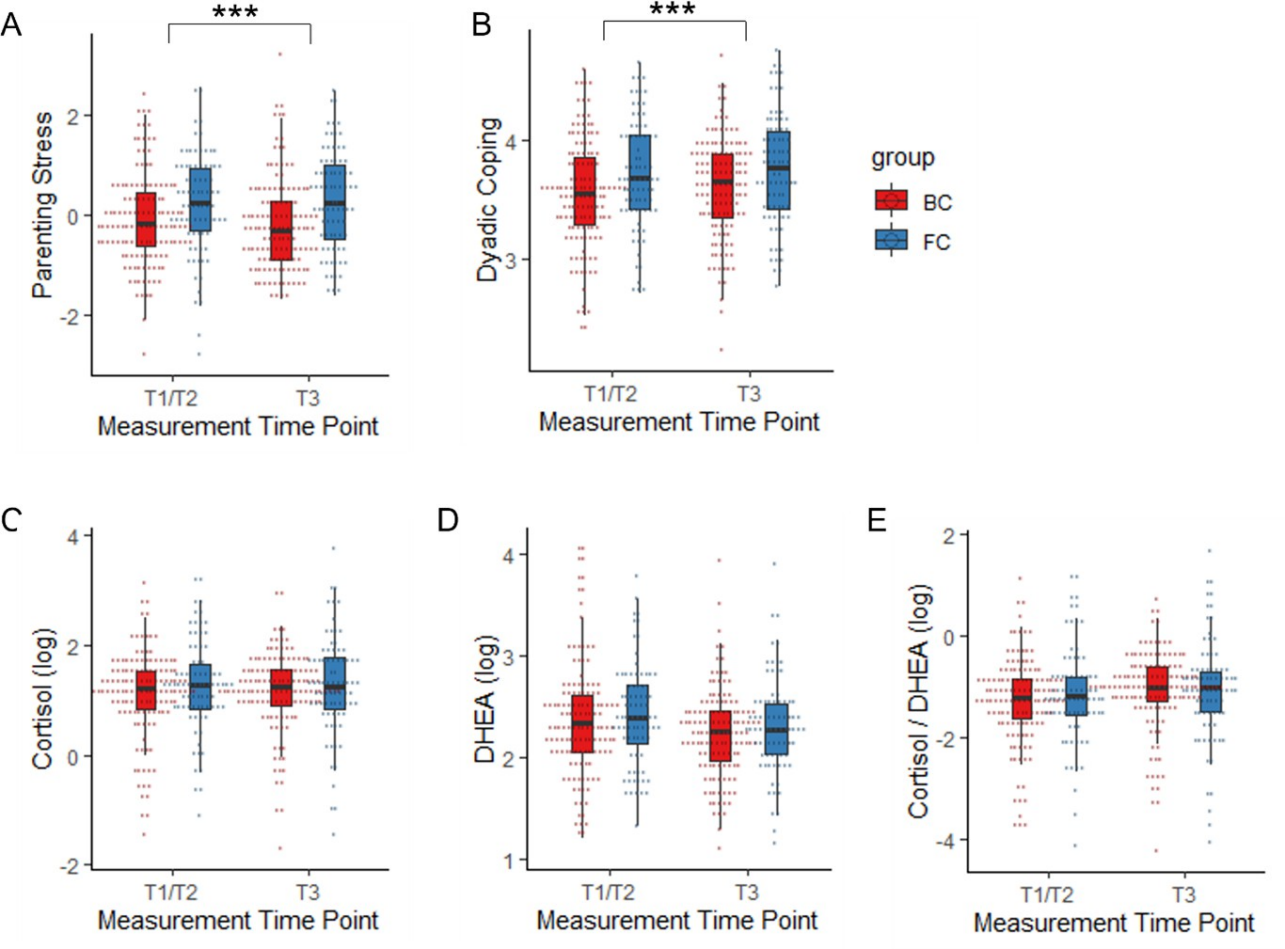
Group differences between foster and biological mothers in study variables. Differences between foster and biological mothers in A) parenting stress, B) dyadic coping, C) cortisol, D) DHEA and E) Cortisol / DHEA per measurement time point. BC = biological control group, FC = foster care group. * *p* < .05. ** *p* < .01. *** *p* < .001.

Both perceived parenting stress and DC were highly stable from T1 to T3 (parenting stress: *r* = .73, *p* < .001, *p*_*adj*._ < .001; DC: *r* = .70, *p* < .001, *p*_*adj*._ < .001). As depicted in Fig 1, foster mothers reported higher levels of perceived parenting stress (*F* (1, 206.05) = 12.007, *p* < .001, *p*_*adj*._ < .001) and more DC (*F* (1, 206.01) = 11.933, *p* < .001, *p*_*adj*._ < .001) compared to biological mothers across time (S3 Table, model 1 and model 2). Parenting stress and DC were negatively correlated with small effect sizes at T1 (foster care group (FC): *r* = -.32, *p* = .005, *p*_*adj*_ = .006; biological control group (BC): *r* = -.24, *p* = .005, *p*_*adj*_ = .006) and at T3 (FC: *r* = -.29, *p* = .017, *p*_*adj*_ = .017; BC: *r* = -.26, *p* = .003, *p*_*adj*_ = .006). This indicated that higher levels of perceived parenting stress were related to lower levels of DC. To further test the longitudinal relationships, stepwise regression analyses indicated that more DC at T1 predicted lower parenting stress at T3 (*ß* = -.137, *p* = .006, *p*_*adj*_ = .012) but not vice versa (*ß* = -.030, *p* = .57, *p*_*adj*_ = .57; Tables 2 and S4). The effect of DC on parenting stress did not significantly differ per group (no interaction with group; Table 2).

**Table 2:** Stepwise regression predicting parenting stress at T3.

	Model 1			Model 2			Model 3		
Variable	*B*	*SE B*	*β*	*B*	*SE B*	*β*	*B*	*SE B*	*Β*
Mother’s age	.004	.008	.028	.002	.008	.013	.002	.008	.013
Child’s age	-.019	.032	-.030	-.008	.032	-.012	-.009	.032	-.014
Group	.147	.111	.074	.232	.114	.116*	.239	.114	.120*
PS T1	.762	.051	.719***	.722	.052	.682***	.720	.052	.680***
DC T1				-.265	.096	-.137**	-.210	.119	-.108
Group x DC T1							-.149	.189	-.047
*R* ^*2*^	.539			.553			.552		
*F* for change in *R*^*2*^	61.971***			7.684**			.619		

*Note*. Model 1 predictors include group and parenting stress (T1), model 2 predictors include group, parenting stress (T1) and dyadic coping (T1) while model 3 predictors include group, parenting stress (T1), dyadic coping (T1) and the group x dyadic coping (T1) interaction, in addition to relevant covariates. Mother’s and child’s age are measured in years at T1 and T1, respectively. Group: 0 = biological control group, 1 = foster care group. DC: Dyadic coping. PS: Parenting stress.

* *p* < .05.

** *p* < .01.

*** *p* < .001.

### 3.2 Group comparisons in hair steroid hormones

Stabilities of hair steroid hormone concentrations were low to moderate (cortisol: *r* = .10, *p* = .13; DHEA: *r* = .40, *p* < .001, *p*_*adj*._ < .001; cortisol / DHEA: *r* = .17, *p* = .013, *p*_*adj*._ = .018). No significant differences were found between foster and biological mothers in cortisol, DHEA and cortisol/DHEA concentrations (Fig 1 and Table 3, model 1) and no time x group interaction (Fig 1 and Table 3, model 2). To calculate the strength of evidence for the absence of between-group differences in hormone levels between foster and biological mothers across timepoints, a Bayes factor of *B* = 0.36 for cortisol, *B* = 0.38 for DHEA and *B* = .30 for cortisol/DHEA was found, showing that there was indicative evidence for cortisol and DHEA as well as substantial evidence for cortisol/DHEA in favor of the null hypothesis.

**Table 3:** Fixed effects of time, group and relevant covariates predicting mother’s hair steroid hormone concentrations.

	Cortisol		DHEA		Cortisol/DHEA	
	Model 1	Model 2	Model 1	Model 2	Model 1	Model 2
Intercept	1.156 (.066)***	1.148 (.072)***	2.366 (.043)***	2.367 (.046)***	-1.207 (.074)***	-1.225 (.080)***
Time	.003 (.006)	.004 (.008)	-.012 (.003)***	-.013 (.004)**	.015 (.007)*	.018 (.009)*
Group	.025 (.099)	.047 (.122)	.063 (.070)	.060 (.079)	-.059 (.112)	-.015 (.137)
Time x Group		-.004 (.013)		.001 (.007)		-.008 (.014)
*Covariates*						
Mother’s age	.011 (.007)	.011 (.007)	-.004 (.005)	-.004 (.005)	.016 (.008)	.016 (.008)
Child’s age	-.057 (.029)	-.057 (.029)	.014 (.021)	.014 (.021)	-.074 (.033)*	-.074 (.033)*
Mother’s BMI			.015 (.006)*	.015 (.006)*		

*Note*. Models are reported for the fixed effects of time and group (model 1) as well as for the fixed effects of time, group and the time x group interaction (model 2), in addition to relevant covariates. Fixed effect estimates are presented with the standard errors in parenthesis. Mother’s and child’s age are measured in years at T1. BMI: Body Mass Index. Time: Time elapsed since T1 in months. Group: 0 = biological control group, 1 = foster care group.

* *p* < .05.

** *p* < .01.

*** *p* < .001.

Because some degree of chronicity may be required for stress to affect the HPA axis, we additionally examined the influence of the duration of children’s foster care placement. No significant correlations were found between time in foster care and cortisol, DHEA and cortisol/DHEA concentrations of foster mothers (all *ps* > .31). Further, findings were consistent (no group effect, no group x time interaction) when including children’s CBCL scores as fixed effect in the linear mixed models (S5 Table). Children’s CBCL score did not predict (foster) mothers’ cortisol, DHEA or cortisol/DHEA concentrations in the models (S4 Table).

### 3.3 Associations of hair steroid hormones to parenting stress and DC

In an exploratory analysis, partial Pearson correlations between study variables, controlling for child’s age, mother’s age, mother’s BMI (for analyses with DHEA), were performed for each group and measurement time point separately (S6 Table). No significant correlations were found between hair steroid hormone concentrations and parenting stress or DC, with the exception of a positive correlation between DC and DHEA (*r* = .29, *p* = .011, *p*_*adj*._ = .264) at T1 for the foster care group which was however not found for T3 and did not survive multiple comparison correction of *p*-values. Consistently, maternal hair cortisol, DHEA and cortisol/DHEA concentrations were not significantly predicted by self-reported parenting stress and DC in the linear mixed models, neither individually (Table 4, model 1) nor in their interaction (Table 4, model 2). Taken together, these results indicated that differences in perceived parenting stress and DC between foster and biological mothers were not, or not strongly, reflected in differences in endocrine markers.

**Table 4:** Fixed effects of parenting stress, dyadic coping and relevant covariates predicting mother’s hair steroid hormone concentrations.

	Cortisol		DHEA		Cortisol/DHEA	
	Model 1	Model 2	Model 1	Model 2	Model 1	Model 2
Intercept	1.175 (.068)***	1.182 (.069)***	2.372 (.045)***	2.377 (.045)***	-1.194 (.076)***	-1.194 (.077)***
Time	.002 (.006)	.002 (.006)	-.013 (.003)***	-.012 (.003)***	.015 (.007)*	.015 (.007)*
Group	-.019 (.105)	-.021 (.105)	.052 (.074)	.050 (.074)	-.092 (.119)	-.092 (.119)
PS	.076 (.046)	.084 (.047)	.014 (.030)	.020 (.031)	.068 (.052)	.068 (.053)
DC	.024 (.089)	.021 (.090)	.017 (.058)	.013 (.058)	-.003 (.100)	-.003 (.101)
PS x DC		.065 (.088)		.053 (.055)		.000 (.099)
*Covariates*						
Mother’s age	.012 (.007)	.013 (.007)	-.003 (.005)	-.003 (.005)	.017 (.009)	.017 (.009)
Child’s age	-.059 (.029)*	-.059 (.029)*	.013 (.021)	.013 (.021)	-.075 (.033)*	-.075 (.033)*
Mother’s BMI			.016 (.006)*	.015 (.006)*		

*Note*. Models are reported for the fixed effects of time, group, parenting stress and dyadic coping (model 1) as well as for the fixed effects of time, group, parenting stress, dyadic coping and the parenting stress x dyadic coping interaction (model 2), in addition to relevant covariates. Fixed effect estimates are presented with the standard errors in parenthesis. Mother’s and child’s age are measured in years at T1. BMI: Body Mass Index. Time: Time elapsed since T1 in months. Group: 0 = biological control group, 1 = foster care group. DC: Dyadic coping. PS: Parenting stress.

* *p* < .05.

** *p* < .01.

*** *p* < .001.

## 4. Discussion

Previous research using the same sample indicated that foster parents reported higher levels of parenting stress and more overall DC in their partnership compared to biological parents [2,3,7]. Here, we asked whether such differences were reflected in objectively measurable markers of long-term HPA axis activity. No significant group differences were found between foster and biological mothers in cortisol, DHEA and cortisol/DHEA concentrations in hair. Further, while DC was negatively related to parenting stress over time, no significant relationships were found to endocrine markers, except for a positive association between DC and DHEA at T1 in the foster care group. More internalizing and externalizing behavior problems of children in foster care likely led to increased levels of parenting stress in foster compared to biological mothers [2,3]. Yet, despite these differences, foster mothers have been found to show better DC [7]. One possible explanation for this is that foster parents already had increased DC competencies before they decided to take care of the child (e.g., due to the selection criteria of the youth welfare services, additional training or experiences related to the foster care process). Another possibility is that they may expect their child to show more challenging behaviors which in turn may lead to increased DC. Further, high DC may be more strongly observed in situations where couples are challenged in their role as parents (for a more detailed discussion see [7]).

The negative relationship between DC and parenting stress builds upon previous findings in children with impairing mental disorders, such as ASD [8], and is consistent with theoretical models that postulate that DC allows parents to jointly cope with everyday stressors. Yet so far, the directionality of effects has been unclear. Here, we found evidence that higher DC within the parent’s partnership was linked to a reduced parenting stress approximately 11 months later, a pattern consistent across both study groups. Contrarily, we did not observe a significant impact of parenting stress on DC. This indicates that DC may be an important relational resource in foster and biological families which allows them to better cope with challenging child behaviors. Yet, intervention studies that are designed to promote positive DC are needed to corroborate this.

Despite differences in DC and parenting stress, foster and biological mothers did not significantly differ in endocrine stress markers, which was mostly supported by our Bayes factor analysis in favor of the H0. It may be hypothesized that higher levels of parenting stress and more DC led to countervailing effects, resulting in the absence of group differences. While the first may be associated with an increased HPA axis activity, the second may buffer some of the stress effects. Yet, since we did not find any significant main or interaction effects between DC and parenting stress on any of the chronic stress markers in our linear mixed models, our results are not consistent with this hypothesis.

Previous studies have found elevated levels of DHEA to be a potential resilience biomarker, as noted in youth residential caregivers [25] and other clinical and community populations [10,38]. This is in line with the observed positive correlation between DC and DHEA within the foster care cohort at T1, which, however, did not survive *p*-value correction. In addition to potential multiple comparison issues, the lack of a similar correlation at T3 questions the robustness of these findings and requires a cautious interpretation. It may be speculated that this is related to the duration of foster care placement and early adaptation processes to the new family constellation. However, foster children already lived for *M* = 17.62 months in their current foster family at T1 and duration of foster care placement did not significantly affect endocrine markers. Despite this, future studies may explore the development of DHEA levels and their association to protective factors, such as DC, in foster parents before and more directly after children’s foster care placement.

The low correspondence between self-reported parenting stress and hair cortisol concentrations is consistent with the current literature, e.g., no consistent associations were found to self-reports of perceived stress or maternal prenatal psychological distress in a meta-analysis and in a systematic review, respectively [14,39]. In line thereof, [40] found higher hair cortisol with increased age but no associations to measures of illbeing (stress, depression, anxiety) and well-being (subjective happiness, life satisfaction, psychological wellbeing) in healthy young females and only to measures of well-being in older females (*M* = 78.6 years). Thus, these findings indicate that hair cortisol concentrations may be poorly reflective of perceived stress and partnership support in healthy populations but could be more strongly related to other physiological adaptations, e.g., with respect to age [40] or BMI [41].

Of note are also the low to moderate 11-month stabilities of hair cortisol and DHEA in the current sample. The reported stabilities vary in the literature mostly from moderate (e.g., *r* = .33 hair cortisol measured one-year apart in N = 79 adults [42]) to high (e.g., *r* = .73 hair cortisol measured one year apart in *N* = 45 adults [43]). Thus, more research is needed investigating the reliability of the measures and differentiating between trait and state components.

The low correspondence between subjective and objective HPA axis stress markers however should not be interpreted as the absence of parenting stress in the current sample. Besides possible measurement issues, both in terms of self-reports and hormonal levels, it could also suggest that significant alterations in hair hormone concentrations require chronic stress exposure above a certain intensity level, which accumulates and eventually exhausts the regulatory capacities of the HPA axis (for a more detailed discussion see [14]). In this regard, it should also be noted that with a mean CBCL *T*-score of ~ 53 in the foster care group and ~ 50 in the biological comparison group (T1), most of the children did not show any emotional and behavioral problems in the clinical range. Thus, although parenting stress was heightened in foster parents, it may, on average, still be lower than in parents with children with impairing mental disorders (see also [44]). Yet, children were relatively young (aged 2–7 years at T1) and some of the problems may become exacerbated at school age or in adolescence when children are faced with increasing demands and developmental tasks. Therefore, longitudinal studies extending over longer periods of time are needed to investigate child behavioral problems and associated parenting stress across different developmental periods and their relationship with HPA axis functioning.

Further, while no relationship was observed with hair hormone concentrations, stress may impact health via various routes, such as by inducing immune dysregulation [45]. Additionally, there may be complex, bidirectional interactions between immune system and HPA axis. For instance, an inverse relationship between the cortisol to C-reactive protein ratio and DC has been found but no relationships to cortisol alone [20]. Therefore, elevated levels of perceived parenting stress in foster and also biological parents should be taken seriously, and interventions should be offered early to prevent long-term stress exposure that can lead to stress-related disorders.

This study is the first that we know of to examine stress and resilience hormone concentrations in foster mothers compared to biological mothers. A particular strength of the study is that several indicators of long-term HPA axis activity (cortisol, DHEA, cortisol/DHEA), self-reported parenting stress and DC were examined longitudinally over approximately one year. Nevertheless, some limitations should be noted. First, while hair hormone concentrations reflect the cumulative concentrations of the preceding three months, parenting stress and DC were measured as they are “typically” perceived in the questionnaires. A closer correspondence may be achieved when stress levels are sampled continuously over the study period [46]. Further, since hair hormone assessments are often only moderately correlated with salivary measurements (e.g., when taking the three-day average of single-day salivary level [47]), findings obtained from hair measurements may not be transferable to other types of hormonal assessments. Salivary cortisol measurements could for instance more accurately indicate stress for short periods in the past [48] or could be used to assess dynamic cortisol responses to acute stressors, e.g., experimental manipulations of stress. Second, possible self-selection effects of the sample need to be considered. Because study participation in the longitudinal study with three assessments was relatively time consuming, families with high stress levels may have been less likely to take part. Third, some of the confounds (e.g., BMI) were only assessed in a subsample of parents. However, since values were completely missing at random, multiple imputation is an effective way to deal with this.

Despite these limitations, our findings add to the growing body of literature showing no or little associations between self-reported measures of stress and social support on the one hand and endocrine markers on the other. However, given the sparsity of research on DHEA, more work is needed to better understand the potential relation of DHEA to (foster) parent’s DC and resilience to stress. In addition, our findings point to the importance of DC in foster and biological families as a relationship resource to more effectively deal with stressors related to raising their child. If these relationships are confirmed, including modules on DC in parent training programs for foster families may reduce parenting stress.

## Supporting information

S1 Table Fixed effects of time, intervention group and relevant covariates predicting stress, dyadic coping and hair steroid hormones. (PDF)

S2 Table Descriptive information on raw hair cortisol, DHEA and cortisol/DHEA values for the foster care group and biological control group. (PDF)

S3 Table Fixed effects of time, group and relevant covariates predicting parenting stress and dyadic coping. (PDF)

S4 Table Stepwise regression predicting dyadic coping at T3. (PDF)

S5 Table Fixed effects of time, group and relevant covariates predicting mother’s hair steroid hormone concentrations controlling for child’s CBCL score. (PDF)

S6 Table Cross-sectional Partial Pearson correlations of the study variables. (PDF)

S1 Text R packages for statistical analyses and their implementation details. (PDF)
