# Supplementary material for: Parenting stress, dyadic coping and endocrine markers of stress and resilience in foster and biological mothers
Source: PLoS One. 2024 Sep 10;19(9):e0310316. doi: 10.1371/journal.pone.0310316 (PMC11386427; doi:10.1371/journal.pone.0310316)
Supplement: S1 Table — (PDF) [file pone.0310316.s001.pdf]

S1 Table. Fixed effects of time, intervention group and relevant covariates predicting parenting stress, dyadic coping and hair steroid hormones.

	Parenting Stress	Dyadic Coping	Cortisol	DHEA	Cortisol/DHEA
Intercept	.313 (.174)	3.777 (.089)***	1.063 (.138)***	2.435 (.085)***	-1.378 (.158)***
Time	.010 (.007)	.004 (.003)	.006 (.012)	-.011 (.005)*	.014 (.013)
Intervention	-.145 (.226)	-.006 (.115)	.115 (.153)	-.041 (.105)	.162 (.188)
Covariates					
Mother's age	-.008 (.017)	-.006 (.009)	.024 (.012)*	-.004 (.008)	.030 (.015)*
Child's age	.078 (.075)	-.002 (.038)	-.022 (.051)	.012 (.035)	-.041 (.062)
Mother's BMI				.017 (.012)	

Note. Fixed effect estimates are presented with the standard errors in parenthesis. Mother's and child's age are measured in years at T1. Time: time elapsed since T1 in months. Intervention: 0 = foster mothers in intervention group, 1 = foster mothers in control group. * $p < .05$. ** $p < .01$. *** $p < .001$.
