# Supplementary material for: Parenting stress, dyadic coping and endocrine markers of stress and resilience in foster and biological mothers
Source: PLoS One. 2024 Sep 10;19(9):e0310316. doi: 10.1371/journal.pone.0310316 (PMC11386427; doi:10.1371/journal.pone.0310316)
Supplement: S2 Table — (PDF) [file pone.0310316.s002.pdf]

S2 Table. Descriptive information on raw hair cortisol, DHEA and cortisol/DHEA values for the foster care group and biological control group.

	Raw		Log-transformed	
	BC	FC	BC	FC
Cortisol				
T1 (*M, SD*)	4.16 (3.66)	5.44 (5.24)	1.08 (0.90)	1.28 (0.95)
T3 (*M, SD*)	4.09 (3.04)	5.95 (6.94)	1.15 (0.80)	1.29 (1.06)
DHEA				
T1 (*M, SD*)	12.70 (9.89)	12.87 (7.70)	2.35 (0.59)	2.41 (0.53)
T3 (*M, SD*)	10.37 (6.16)	11.79 (7.58)	2.22 (0.47)	2.32 (0.54)
Cortisol/DHEA				
T1 (*M, SD*)	.43 (.46)	.57 (.71)	-1.30 (1.01)	-1.12 (1.09)
T3 (*M, SD*)	.47 (.36)	.69 (.93)	-1.08 (.91)	-1.04 (1.21)

Note. BC: biological control group. FC: foster care group. Sample description is based on data prior to multiple imputation. Both raw data and log-transformed data are presented.
