# Supplementary material for: Parenting stress, dyadic coping and endocrine markers of stress and resilience in foster and biological mothers
Source: PLoS One. 2024 Sep 10;19(9):e0310316. doi: 10.1371/journal.pone.0310316 (PMC11386427; doi:10.1371/journal.pone.0310316)
Supplement: S4 Table — (PDF) [file pone.0310316.s004.pdf]

S5 Table. Stepwise regression predicting dyadic coping at T3.

	Model 1			Model 2			Model 3		
Variable	*B*	*SE B*	*β*	*B*	*SE B*	*β*	*B*	*SE B*	*β*
Mother's age	-.008	.004	-.116*	-.009	.004	-.121*	-.009	.004	-.120*
Child's age	-.026	.016	-.087	-.026	.016	-.084	-.025	.016	-.081
Group	.085	.055	.089	.094	.058	.099	.097	.058	.101
DC T1	.646	.047	.691***	.639	.048	.683***	.637	.049	.682***
PS T1				-.015	.026	-.030	.000	.033	-.001
Group x PS T1							-.038	.052	-.046
R^2	.507			.505			.504		
F for change in R^2	54.666***			.330			.530		

Note. Model 1 predictors include group and dyadic coping (T1), model 2 predictors include group, dyadic coping (T1) and parenting stress (T1) while model 3 predictors include group, dyadic coping (T1), parenting stress (T1) and the group x parenting stress (T1) interaction, in addition to relevant covariates. Mother's and child's age are measured in years at T1. DC: dyadic coping. PS: Parenting stress. Group: 0 = biological control group, 1 = foster care group. * $p < .05$. ** $p < .01$. *** $p < .001$.
