# Supplementary material for: Parenting stress, dyadic coping and endocrine markers of stress and resilience in foster and biological mothers
Source: PLoS One. 2024 Sep 10;19(9):e0310316. doi: 10.1371/journal.pone.0310316 (PMC11386427; doi:10.1371/journal.pone.0310316)
Supplement: S5 Table — (PDF) [file pone.0310316.s005.pdf]

S6 Table. Fixed effects of time, group and relevant covariates predicting mother's hair steroid hormone concentrations controlling for child's CBCL score.

	Cortisol		DHEA		Cortisol/DHEA	
	Model 1	Model 2	Model 1	Model 2	Model 1	Model 2
Intercept	1.162 (.069)***	1.151 (.075)***	2.374 (.046)***	2.379 (.048)***	-1.214 (.078)***	-1.236 (.084)***
Time	.001 (.007)	.003 (.008)	-.013 (.003)***	-.014 (.004)***	.014 (.007)*	.018 (.009)*
Group	.051 (.110)	.079 (.131)	.065 (.078)	.052 (.085)	-.024 (.127)	.033 (.147)
Group x Time		-.005 (.013)		.003 (.007)		-.011 (.014)
Covariates						
Mother's age	.012 (.008)	.012 (.008)	-.003 (.006)	-.003 (.006)	.016 (.009)	.017 (.009)
Child's age	-.054 (.033)	-.054 (.033)	.013 (.023)	.013 (.023)	-.072 (.038)	-.073 (.038)
Mother's BMI			.016 (.007)*	.016 (.007)*		
Child's CBCL	-.003 (.005)	-.003 (.005)	-.001 (.003)	-.001 (.003)	-.002 (.005)	-.002 (.005)

Note. Models are reported for the fixed effects of time and group (model 1) as well as for the fixed effects of time, group and the time x group interaction (model 2), in addition to relevant covariates and child's CBCL score. Fixed effect estimates are presented with the standard errors in parenthesis. Mother's and child's age are measured in years at T1. BMI: Body Mass Index. CBCL: total score of the Child Behavior Checklist (*T*-value). Time: time elapsed since T1 in months. Group: 0 = biological control group, 1 = foster care group. * $p < .05$. ** $p < .01$. *** $p < .001$.
