# Supplementary material for: Parenting stress, dyadic coping and endocrine markers of stress and resilience in foster and biological mothers
Source: PLoS One. 2024 Sep 10;19(9):e0310316. doi: 10.1371/journal.pone.0310316 (PMC11386427; doi:10.1371/journal.pone.0310316)
Supplement: S6 Table — (PDF) [file pone.0310316.s006.pdf]

S7a Table. Cross-sectional Partial Pearson correlations of the study variables at T1 for the biological control group.

	Cortisol	DHEA	Cortisol/DHEA	Parenting Stress	Dyadic Coping
Cortisol		.31***	.72***	.05	.05
DHEA			-.43***	.06	-.04
Cortisol/DHEA				.01	.11
Parenting Stress					-.24**
Dyadic Coping					

Note. Control variables include mother's age (T1) and child's age (T1 or T3, respectively). Further, for all correlations with DHEA, mother's BMI (T1 or T3, respectively) was included as an additional control variable. * $p < .05$. ** $p < .01$. *** $p < .001$.

S7b Table. Cross-sectional Partial Pearson correlations of the study variables at T3 for the biological control group.

	Cortisol	DHEA	Cortisol/DHEA	Parenting Stress	Dyadic Coping
Cortisol		.13	.82***	.12	-.06
DHEA			-.45***	-.06	.01
Cortisol/DHEA				.15	-.06
Parenting Stress					-.26**
Dyadic Coping					

Note. Control variables include mother's age (T1) and child's age (T1 or T3, respectively). Further, for all correlations with DHEA, mother's BMI (T1 or T3, respectively) was included as an additional control variable. * $p < .05$. ** $p < .01$. *** $p < .001$.

S7c Table. Cross-sectional Partial Pearson correlations of the study variables at T1 for the foster care group.

	Cortisol	DHEA	Cortisol/DHEA	Parenting Stress	Dyadic Coping
Cortisol		.10	.85***	.15	.01
DHEA			- .44***	.06	.29*
Cortisol/DHEA				-.11	-.15
Parenting Stress					-.32**
Dyadic Coping					

Note.T1 Control variables include mother's age (T1) and child's age (T1 or T3, respectively). Further, for all correlations with DHEA, mother's BMI (T1 or T3, respectively) was included as an additional control variable. * $p < .05$. ** $p < .01$. *** $p < .001$.

S7d Table. Cross-sectional Partial Pearson correlations of the study variables at T3 for the foster care group.

	Cortisol	DHEA	Cortisol/DHEA	Parenting Stress	Dyadic Coping
Cortisol		-.03	.89***	-.03	.02
DHEA			-.48***	-.02	-.01
Cortisol/DHEA				-.01	.02
Parenting Stress					-.29*
Dyadic Coping					

Note. Control variables include mother's age (T1) and child's age (T1 or T3, respectively). Further, for all correlations with DHEA, mother's BMI (T1 or T3, respectively) was included as an additional control variable. * $p < .05$. ** $p < .01$. *** $p < .001$.
