# Supplementary material for: Parenting stress, dyadic coping and endocrine markers of stress and resilience in foster and biological mothers
Source: PLoS One. 2024 Sep 10;19(9):e0310316. doi: 10.1371/journal.pone.0310316 (PMC11386427; doi:10.1371/journal.pone.0310316)
Supplement: S1 Text — (PDF) [file pone.0310316.s007.pdf]

S3 Text. R packages for statistical analyses and their implementation details.

Multiple imputations were conducted in the R package 'mice' [1]. Subsequently, imputed data sets were merged into a single data set by replacing missing values with the mean of all imputed values using the R package 'sjmisc' [2].

Linear mixed model analyses were conducted with the R package 'lme4' [3]. Final models were fitted by restricted maximum likelihood (REML) and *p*-values were obtained using the summary function of the R package 'lmerTest' [4] with the Satterthwaite approximation for the degrees of freedom.

Bayesian analyses were conducted using 'bayes_factor' from the 'brms' R package [5]. Bayesian models were fitted with three chains and 10000 iterations, using the brms default priors, which are weakly informative. Skew normal response functions were chosen since they provided better model fits than Gaussian response functions, as indicated by lower leave-one-out indices (LOO, [6]). Finally, convergence of the models was checked using the Gelman-Rubin statistic R and effective sample size (ESS) as well as by visually inspecting the trace plots for funnels.

False Discovery Rate (FDR) correction of *p*-values was performed for each research question and analysis type using the R command 'p.adjust' and the procedure by [7]. Specifically, for RQ1, *p*-values were adjusted for four correlations and two multiple regressions, for RQ2, for three linear mixed models and for RQ3, for 24 correlations and three linear mixed models. In case of significant effects, both adjusted (p_{adj}) and unadjusted *p*-values are reported.

References

1. van Buuren S, Groothuis-Oudshoorn K. mice: Multivariate imputation by chained equations in R. J Stat Softw. 2011; 45 (3): 1–67. doi: 10.18637/jss.v045.i03
2. Lüdecke, D. sjmisc: Data and variable transformation functions. J Open Source Softw. 2018; 3 (26), 754. doi: 10.21105/joss.00754
3. Bates D, Mächler M, Bolker B, Walker S. Fitting linear mixed-effects models using lme4. J Stat Softw. 2014; 67(1). doi: 10.18637/jss.v067.i01
4. Kuznetsova A, Brockhoff PB, Christensen RHB. lmerTest package: Tests in linear mixed effects models. J Stat Softw. 2017; 82 (13): 1–26. doi: 10.18637/jss.v082.i13
5. Bürkner P-C. brms: An R package for Bayesian multilevel models using Stan. J Stat Softw. 2017; 80 (1): 1–28. doi: 10.18637/jss.v080.i01
6. Vehtari A, Gelman A, Gabry J. Practical Bayesian model evaluation using leave-one-out cross-validation and WAIC. Stat Comput. 2017; 27: 1413-1432. doi: 10.1007/s11222-016-9696-4

7. Benjamini Y, Hochberg Y. Controlling the false discovery rate: a practical and powerful approach to multiple testing. J R Stat Soc Series B. 1995; 57, 289-300. doi: 10.1111/j.2517-6161.1995.tb02031.x
